# Event and Comment

**Published:** 1914-12-15

**Authors:** 


### DENTAL REGISTER.

**Vol. LXIX. December 15, 1914. No. 12**

### EVENT AND COMMENT.

NATIONAL ASSOCIATION BULLETIN. The October number of the *Bulletin* comes to us in full fledged journal form. Its artistic cover page and its 175 pages of reading matter include reports of the annual meeting besides notices and eight pages of editorial matter. For a mere "bulletin" it certainly puts up a good appearance. One would scarcely know it from a regular Dental Journal, except that it does not carry a single page of advertising matter. It contains a large number of cuts, illustrating articles and some inserts; one of President Gallie and some colored illustrations of Dr. Hartzel's paper which are very fine. We don't see how it is to be kept up with the one dollar membership, but we presume other numbers will not have so many pages. Dr. King starts out very promisingly as an editor and he shows all the signs of making good. It will be interesting to see how long this journal will last without accepting advertising. We don't know of but one professional journal that is able to publish creditably without advertising. If any journal can do this we hope the *National Bulletin* may be that journal. We recently saw a circular letter from the business manager of a journal published by the profession which made an earnest request of all the members of that Association, that they particularly notice a certain advertisement and send orders to this concern, as a year's contract for advertising depended on the response to that particular advertisement. Does that look as though the profession was supporting its own journal? The truth of the matter is that very few dental journals, or any other kind, in fact, would be printed if it were not for the advertising patronage. The publisher of one of our popular dental journals told me that it cost his house one dollar and fifty cents to print the journal which he was selling for one dollar. Now who gets the benefit, the advertiser, the publisher or the reader? The advertiser must know it is good business or he would'nt pay good money merely to keep the journal going. The publisher must get his remuneration from some one. The reader seems to be the recipient of a bargain to say the least. And yet when we trace the matter back, the reader will be found, in the last analysis, to be holding the bag. He not only pays his subscription but he pays the profits to the dealer which enable him to publish the journal.

Fortunately the dentist pays his money out so gradually that it never seems a burden. He gets much out of his literature that has no tangible values. He does not turn all the knowledge he obtains from this source into money, at least not right away, He gets a certain amount of culture from careful and systematic reading that is invaluable. It keeps him in touch with the general activities of his profession, which causes him to have a broader sympathy with it, and makes him careful of its good name, and it also interests him in all other persons who are practicing his profession. It is a fine thing to know the men in the profession who are doing things and telling others, through the journals, what they have discovered. It is like getting a letter from home to read the contributions of our friends and the men whom we perhaps know only by their contributions to our journals. Let every body then boost all the journals he believes worthy.

### NATIONAL DENTAL RESEARCH FOUNDATION.

The work of organizing and carrying through the first year of this undertaking is reported in the *National Bulletin* for October, and the reports of most of the research workers on work done is printed in the same journal. The profession is to be congratulated that this work has been so successfully begun. Up to July 6, 1914, $7,067.50 were collected, and $4,378.06 were expended. Much of the credit for this successful result is due to the untiring endeavor of the chairman of the commission, Dr. Weston A. Price, who has labored tremendously to secure the money and to get the research men who would undertake the various problems. Dr. Price has contributed the first thousand dollars towards a permanent endowment of the commission and he hopes before long to be able to announce other contributions to the cause. More money should be contributed by the profession on the basis of a five year's contract, in order that the work may be properly enlarged to include many other subjects not yet undertaken, and to carry on to some definite conclusion those already well under way. It is to be hoped none will be disappointed if immediate results are not forthcoming, as all these problems are difficult and much time will necessarily be consumed in carrying out experiments that will not demonstrate positive results at once.

